# Does hydration status affect ventilation during prolonged exercise?

**DOI:** 10.1113/EP092332

**Published:** 2025-01-01

**Authors:** Naoto Fujii

**Affiliations:** ^1^ Faculty of Health and Sport Sciences University of Tsukuba Tsukuba Japan; ^2^ Advanced Research Initiative for Human High Performance (ARIHHP) University of Tsukuba Tsukuba Japan

**Keywords:** control of breathing, endurance performance, hypohydration

## 1 INTRODUCTION

Connections link a sequence of three related research papers. The central article which links the other two papers has been published in Experimental Physiology. In a Connections article, an author (or authors) of the central article outlines its principal novel findings, tracing how they were influenced by the first article and how the central article has contributed to the developments made in the third article. The author(s) may also speculate on the direction of future research in the field. Connections articles aim to set the research in a wide context.

Pulmonary ventilation (hereafter referred to as ventilation) is tightly regulated in response to changes in metabolic rate (i.e., O_2_ consumption and CO_2_ production), maintaining arterial O_2_ and CO_2_ partial pressures. Therefore, it is not surprising that ventilation increases proportionally with exercise intensity and thus metabolic rate. As exercise intensity increases, wherein acidosis is intensified, ventilation increases exponentially relative to metabolic rate such that arterial partial pressures of CO_2_ decrease (hypocapnia) to minimize acidosis. In addition to metabolic rate, elevations in core temperature appear to increase ventilation with concomitant hypocapnia at rest and during prolonged submaximal exercise, particularly in the heat. My colleagues and I in Japan have conducted several experiments to assess this response, which we term hyperthermia‐induced hyperventilation in our recent studies. I believe the underlying mechanisms and physiological significance of this response during exercise in humans remain to be elucidated. Nevertheless, hyperthermia‐induced hyperventilation during prolonged exercise in the heat might occur to maintain homeostasis by: (1) mitigating acidosis through hypocapnia; (2) increasing respiratory heat loss; and (3) increasing blood oxygenation by elevating the arterial partial pressure of O_2_ and by a leftward shift in the haemoglobin–oxygen dissociation curve mediated by hypocapnia. The latter adaptation counteracts the rightward shift of the haemoglobin–oxygen dissociation curve induced by hyperthermia, which compromises oxygen loading to the blood. It should be noted that hyperthermia‐induced hyperventilation leads to: (1) reduced cerebral blood flow owing to hypocapnia; (2) increased oxygen cost of ventilation; and (3) elevated breathing effort, all of which can increase physiological and psychological strain, potentially impairing exercise tolerance and endurance performance. Therefore, I believe that interventions aimed at mitigating hyperthermia‐induced hyperventilation could potentially reduce physiological and psychological strain, thereby enhancing exercise tolerance and performance in the heat.

Fluid loss through sweating often exceeds fluid intake during prolonged exercise in the heat, causing dehydration. The key question is whether dehydration contributes to hyperthermia‐induced hyperventilation during prolonged exercise in the heat. Addressing this question could clarify whether proper hydration attenuates hyperthermia‐induced hyperventilation, thereby improving exercise tolerance and performance in these conditions.

As discussed earlier, ventilation can facilitate heat loss by increasing evaporative and dry heat dissipation. In this context, animals such as dogs and cats increase their ventilation during heat exposure, a response known as panting, serving as their primary heat‐loss response. During exercise with heat stress, panting animals must regulate their ventilation to accommodate both metabolic and thermal demands. In this regard, Baker and Nijland (1993) reported that in goats, severe dehydration (9.2% body mass loss!) reduced respiratory evaporative heat loss (hence body water loss) at given central blood temperatures during treadmill walking with lost weight compensated by weighed saddlebags. Their data suggest an attenuated panting response, which aligns with fluid‐conservation mechanisms.

It should be emphasized that panting animals and humans exhibit distinct differences in heat‐loss responses. Humans have evolved sweating function, enabling prolonged exercise in hot conditions, such as marathon races during the Summer Olympic Games. In contrast, although goats are capable of sweating, this response is short lived owing to sweat gland fatigue. Additionally, the extent of water and heat loss through ventilation in humans is minimal compared with panting animals. Therefore, in humans, although dehydration is known to impair the sweating response as a means to conserve body water, it remains unclear whether dehydration similarly attenuates hyperthermia‐induced hyperventilation.

As part of my master's thesis, I investigated whether dehydration influences hyperthermia‐induced hyperventilation during exercise in the heat in sedentary or physically active male participants who performed both a fluid replacement trial and a no‐fluid replacement trial in random order (Fujii et al., 2008). Participants completed two bouts of cycling exercise (30–60 min) at 50% of peak oxygen uptake in the heat, with a long recovery period between the bouts. During the recovery period in the fluid replacement trial, participants consumed water containing 25 mosmol/L of sodium, whereas no fluids were consumed in the no‐fluid replacement trial. After the first exercise, participants lost 2.5% of their body mass in the no‐fluid replacement trial, whereas the sodium drink successfully restored body fluid levels to a euhydrated state in the fluid replacement trial. Consequently, participants performed the second exercise bout with different hydration levels in each trial. Although the heat‐loss response of cutaneous vasodilatation was attenuated in the no‐fluid trial versus the fluid replacement trial, the slope relating minute ventilation and core temperature, an index of hyperthermia‐induced hyperventilation, remained unaffected. Thus, we conclude that dehydration (2.5% body mass loss before exercise) does not influence hyperthermia‐induced hyperventilation during exercise in humans.

As a follow‐up study, González‐Alonso et al. (2023) reanalysed previous data from studies conducted in Copenhagen, Denmark and Texas, USA. Their participants were endurance‐trained individuals (cyclists) who exercised for a longer duration (∼120 min) at a higher intensity (60%–70% of maximal O_2_ uptake) relative to our study. In addition, the extent of dehydration (∼4% body mass loss) and hyperthermia (∼39.5°C core temperature) was more severe than in our study. Consistent with our findings, they demonstrated that minute ventilation was closely related to core temperature and that dehydration alone did not affect minute ventilation during exercise, because fluid replacement and blood volume restoration had no impact on ventilation. Based on measured blood variables, they found that circulating catecholamines were positively correlated with minute ventilation during exercise in the heat. Interestingly, they also discovered that infusion of adrenaline, a hormone known to mediate increases in body temperature and ventilation at rest, to levels comparable to those observed during high‐intensity exercise, increased both core temperature and minute ventilation during prolonged exercise in the heat. Therefore, sympathoadrenal activation might partly mediate hyperthermia‐induced hyperventilation during prolonged exercise in the heat.

**Connected Articles**

- Baker, M. A., & Nijland, M. J. (1993). Selective brain cooling in goats: effects of exercise and dehydration. *The Journal of Physiology*, *471*, 679–692.
- Fujii, N., Honda, Y., Hayashi, K., Kondo, N., & Nishiyasu, T. (2008). Effect of hypohydration on hyperthermic hyperpnea and cutaneous vasodilation during exercise in men. *Journal of Applied Physiology*, *105*, 1509–1518.
- Gonzalez‐Alonso, J., Calbet, J. A. L., Mora‐Rodriguez, R., & Kippelen, P. (2023). Pulmonary ventilation and gas exchange during prolonged exercise in humans: Influence of dehydration, hyperthermia and sympathoadrenal activity. *Experimental Physiology*, *108*, 188–206.

Based on combined data from our study (Fujii et al., 2008) and González‐Alonso et al. (2023), dehydration does not appear to affect hyperthermia‐induced hyperventilation, as indicated by the linear relationship between minute ventilation and core temperature, during prolonged exercise in the heat in humans. However, dehydration can impair heat‐loss responses, accelerating core temperature elevations, as observed by González‐Alonso et al. (2023), ultimately exacerbating hyperventilation. Therefore, minimizing dehydration through fluid ingestion before and during prolonged exercise in the heat can attenuate hyperventilation by preventing an excessive increase in core temperature, potentially improving exercise tolerance or endurance performance in hot conditions. Likewise, any intervention that reduces core temperature (e.g., heat acclimatization, cooling) could alleviate the physiological and psychological strain associated with hyperthermia‐induced hyperventilation, possibly improving endurance performance in the heat. However, the potential advantages of body weight reduction owing to dehydration should also be considered when evaluating endurance performance in weight‐bearing exercises, such as running.

Lastly, I would like to highlight the knowledge gaps that need to be addressed in future research. We do not yet know whether the aforementioned findings obtained in human studies (Fujii et al., 2008; Gonzalez‐Alonso et al., 2023) apply to severe‐intensity exercise. It should be noted that during severe‐intensity exercise, O_2_ uptake and CO_2_ output drift over time, complicating the distinction between the effects of hyperthermia and metabolic stimuli on ventilation. It also remains uncertain whether similar results would be observed in different populations, such as elite athletes, older adults, children and females. Regarding females, both our study (Fujii et al., 2008) and the study by González‐Alonso et al. (2023) tested males only, because limited attention was given to testing females when the original data in these studies were published [González‐Alonso et al. (2023) reanalysed data initially published between 1996 and 2000].

## AUTHOR CONTRIBUTIONS

Sole author.

## CONFLICT OF INTEREST

None declared.

## FUNDING INFORMATION

None.
